# Bee Venom Phospholipase A2 Alleviate House Dust Mite-Induced Atopic Dermatitis-Like Skin Lesions by the CD206 Mannose Receptor

**DOI:** 10.3390/toxins10040146

**Published:** 2018-04-02

**Authors:** Dasom Shin, Won Choi, Hyunsu Bae

**Affiliations:** Department of Science in Korean Medicine, College of Korean Medicine, Kyung Hee University, 26 kyungheedae-ro, dongdaemoon-ku, Seoul 02447, Korea; ssd060@naver.com (D.S.); wonones@naver.com (W.C.)

**Keywords:** atopic dermatitis (AD), house dust mite extract (DFE), 2,4-dinitrochlorobenzene (DNCB), bee venom phospholipase A2 (bvPLA2), skin inflammation, CD206, mannose receptor

## Abstract

Atopic dermatitis (AD) is a chronic inflammatory skin disease characterized by highly pruritic, erythematous, and eczematous skin plaques. We previously reported that phospholipase A2 (PLA2) derived from bee venom alleviates AD-like skin lesions induced by 2,4-dinitrochlorobenzene (DNCB) and house dust mite extract (*Dermatophagoides farinae* extract, DFE) in a murine model. However, the underlying mechanisms of PLA2 action in actopic dermatitis remain unclear. In this study, we showed that PLA2 treatment inhibited epidermal thickness, serum immunoglobulin E (IgE) and cytokine levels, macrophage and mast cell infiltration in the ear of an AD model induced by DFE and DNCB. In contrast, these effects were abrogated in CD206 mannose receptor-deficient mice exposed to DFE and DNCB in the ear. These data suggest that bvPLA2 alleviates atopic skin inflammation via interaction with CD206.

## 1. Introduction

Atopic dermatitis (AD), also known as atopic eczema, is a chronic inflammatory skin disease associated with intense pruritus [1]. The details of mechanisms underlying AD process are still unclear, although large studies investigated therapeutic targets and pathophysiology [2]. AD affects nearly 20% of children and almost 7% of adults [3,4].

Although it is still disputed, it is generally suggested that AD is a chronic inflammatory disease associated with increased level of proinflammatory cytokines, serum immunoglobulin E (IgE) in the mast cells of the skin [5,6]. Until now, topical corticosteroids have been used as a first-line treatment for AD [7]. Although further studies are needed for validation, novel therapeutic approaches have been discovered, including ceramides and other components involved in epidermal differentiation and maintenance of epidermal barriers [8]. However, most AD therapies have been reported to have side effects, especially in children [9]. Recent advances in herbal medicine suggest potential therapies for inflammatory skin diseases [10].

Bee venom, which has long been used in alternative medicine, has been used to treat asthma, multiple sclerosis, cancer, and rheumatoid arthritis, as a powerful immuno-modulatory agent [11,12,13,14]. Bee venom is composed of various peptides and proteins such as melittin, phospholipase A2, apamin, and mast cell degranulating peptide [15,16,17]. The bee venom phospholipase A2 (bvPLA2), one of the major components of bee venom, plays a central role in the regulation of phospholipid metabolism, signal transduction, inflammatory and immune response [18,19,20]. We recently reported that bvPLA2 acts by activating regulatory T cell (Tregs) in a murine model of allergic asthma and cisplatin-induced nephrotoxicity via CD206 mannose receptor [21,22]. In addition, bvPLA2 suppresses AD-like skin lesions in Balb/c mouse model induced by *Dermatophagoides farinae* extract (DFE) and 2,4-dinitrochlorobenzene (DNCB) [10]. Based on our previous studies, we hypothesized that bvPLA2 has an inhibitory effect on AD-like skin lesions through the CD206 receptor. AD signs and symptoms were evaluated in wild-type or CD206-deficient mice challenged on the ear with DFE and DNCB.

## 2. Results

### 2.1. bvPLA2 Treatment Alleviated DFE/DNCB-Induced Ear Thickness and AD-Like Symptoms

**Figure 1:**
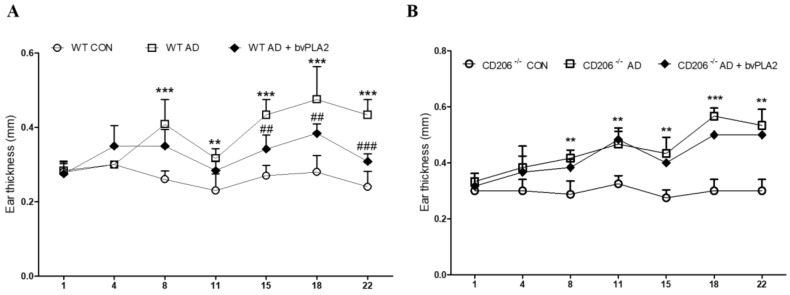
Effect of bvPLA2 on ear thickness of DFE/DNCB-induced AD mice depending on CD206 receptor. Images of the ear skin lesions in groups of mice taken on day 28. Ear thickness was measured using a dial thickness gauge 24 h after DFE/DNCB application for 4 weeks. DFE/DNCB caused ear swelling in wild-type (WT) and CD206^−/−^ mice but bvPLA2 effectively suppressed AD-related ear swelling only in WT mice (**A**), but not CD206^−/−^ mice (**B**). CON: normal control, AD: DFE/DNCB, and AD + bvPLA2: DFE/DNCB + bvPLA2 (80 ng/ear, 20 µL). The data are displayed as the mean ± SD. The statistical analyses were conducted with one-way ANOVA followed by Newman–Keuls multiple comparison tests (*** *p* < 0.001, ** *p* < 0.01 vs. con and **^###^** *p* < 0.001, ^##^ *p* < 0.01 vs. AD; *n* = 3–5).

To investigate the effect of bvPLA2 in DFE/DNCB-induced AD-like skin lesion, bvPLA2 was used to treat mice subjected to DFE/DNCB treatment. As shown in Figure 1, DFE/DNCB induced significant skin swelling on both ears in wild-type (WT) and CD206-deficient (CD206^−/−^) mice. However, bvPLA2 effectively suppressed AD-related skin swelling in WT mice, but not CD206^−/−^ mice.

The ear thickness was evaluated using dial thickness gauge during 4 weeks (Figure 1). In WT mice, DFE/DNCB treatment significantly increased the ear thickness compared with the control group while bvPLA2 improved ear thickness in DFE/DNCB-induced AD mice (Figure 1A). On the other hand, in CD206^−/−^ mice, no significant effect was observed after bvPLA2 treatment (Figure 1B).

### 2.2. bvPLA2 Reduced Serum IgE in DFE/DNCB-Treated Mice

**Figure 2:**
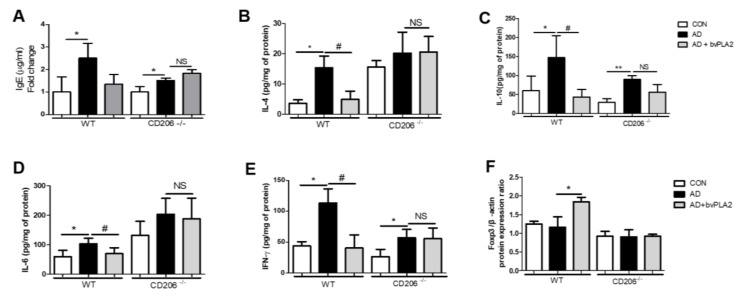
Effects of bvPLA2 on expression of serum total immunoglobulin E (IgE) and cytokines in the ear tissues of DFE/DNCB-induced AD mice via CD206. The total serum IgE levels and the concentration of Th1 and Th2 cytokines were measured by ELISA and Foxp3 expression was observed by western blot. (**A**) IgE; (**B**) Interleukin-4 (IL-4); (**C**) Interleukin-10 (IL-10); (**D**) Interleukin-6 (IL-6); (**E**) Interferon- γ (IFN-γ) and (**F**) Foxp3. The data are shown as the means ± SD. The statistical analyses were conducted with a one-way ANOVA followed by Newman–Keuls multiple comparison tests. CON: normal control, AD: DFE/DNCB treatment, and AD+bvPLA2: DFE/DNCB treatment + bvPLA2 treatment. (** *p* < 0.01, * *p* < 0.05 vs. Control and ^#^ *p* < 0.05, NS vs. AD; *n* = 3–5).

We previously reported the therapeutic effect of bvPLA2 in DFE/DNCB-induced AD mice [10]. In this study, we measured the total IgE from serum following bvPLA2 treatment of WT and CD206^−/−^ mice. As shown in Figure 2A, DEF/DNCB treatment induced a dramatic increase in serum IgE in mice. Interestingly, bvPLA2 treatment effectively inhibited IgE increase in WT mice challenged with DFE and DNCB. In contrast, bvPLA2 treatment showed no significant difference in CD206^−/−^ mice after DFE/DNCB treatment.

### 2.3. bvPLA2 Abrogated AD-Related Th1 and Th2 Cytokine Production via CD206

We evaluated the expression of Th1- and Th2-cytokines using ELISA (Figure 2B–E). Similar to our previous study showing that bvPLA2 was effective in decreasing Th2 cytokine expression in an asthma mouse model [22], treatment of WT mice with bvPLA2 following DFE/DNCB exposure significantly decreased the expression of Th1 and Th2 cytokines including IFN-γ, IL-4, IL-6, and IL-10. The cytokine expression was remarkably blocked by bvPLA2 in WT mice treated with DFE/DNCB. However, bvPLA2 treatment of CD206^−/−^ mice induced no significant variation in expression of Th1 or Th2 cytokines following exposure to DFE/DNCB.

### 2.4. bvPLA2 Is Associated with Treg Induction through the CD206 Receptor

Previous reports have shown that bvPLA2 treatment effectively increased Foxp3-expressing CD4^+^CD25^+^Tregs in AD-like skin lesions induced by DFE/DNCB [11]. To confirm that bvPLA2 induced Treg through CD206, the expression of Foxp3 in the ear tissues were measured using western blot. In WT mice, bvPLA2 treated group showed a significant increase in Foxp3 protein expression compared to AD group, while in CD206^−/−^ mice, bvPLA2 was not effective in inducing Treg (Figure 2F).

### 2.5. bvPLA2 Decreased DFE/DNCB-Induced Epidermal and Dermal Thickness and Infiltration of Inflammatory Cells Depending on CD206

**Figure 3:**
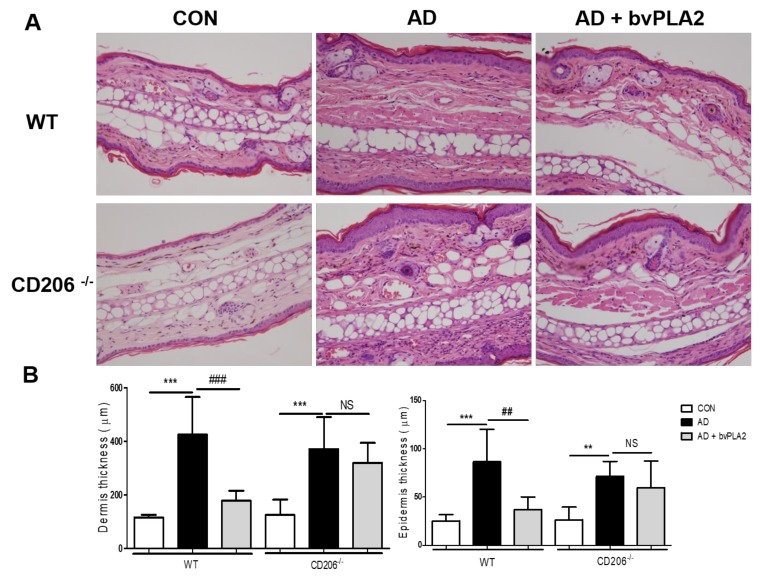
Protective effect of bvPLA2 on the histology of the ear in DFE/DNCB-induced mice via CD206. The ear tissue sections obtained from wild-type (WT) mice (**A**) or CD206-deficient mice (CD206^−/−^ mice) (**A**) were stained with Hematoxylin and Eosin (H&E) (magnification ×200). The thicknesses of dermis and epidermis were quantified based on the H&E stained sections (**B**). CON: normal control, AD: DFE/DNCB treatment, and AD + bvPLA2: DFE/DNCB treatment + bvPLA2 treatment. (** *p* < 0.01, *** *p* < 0.001 versus Control; NS, ^##^ *p* < 0.01, ^###^ *p* < 0.001, versus AD).

**Figure 4:**
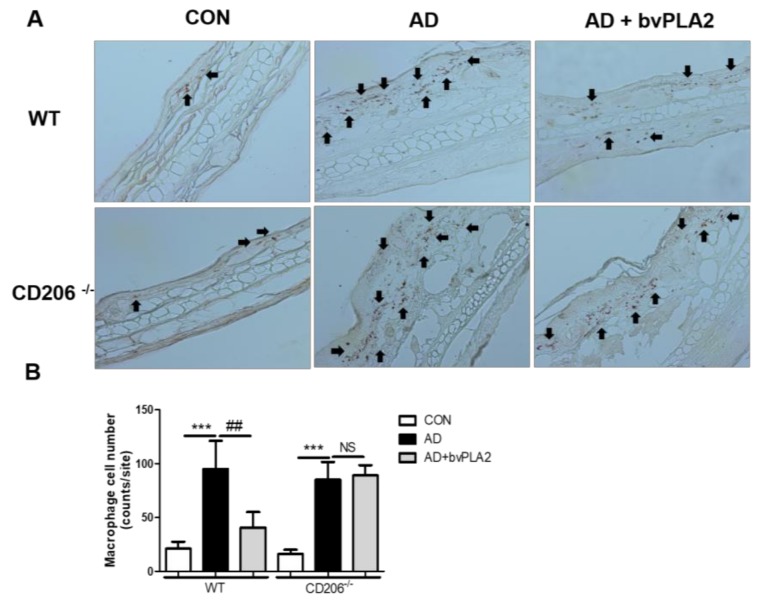
Protective effect of bvPLA2 on macrophage infiltration in DFE/DNCB-induced mice via CD206. The ear tissue sections obtained from wild-type (WT) mice (**A**) or CD206-deficient mice (CD206^−/−^ mice) (**A**) were stained with IHC (magnification ×200). Arrows indicate infiltrated macrophages. The macrophage cell number count graph on the IHC stained sections (**B**). CON: normal control, AD: DFE/DNCB treatment, and AD + bvPLA2: DFE/DNCB treatment + bvPLA2 treatment. (*** *p* < 0.001 versus Control; NS, ^##^ *p* < 0.01 versus AD).

To examine histological changes and macrophage infiltration in the ear tissues, ear tissues were stained with Hematoxylin and Eosin (H&E) (Figure 3) and immunohistochemistry (IHC) (Figure 4). Similar to our previous study, the AD group of WT mice exhibited an increase in epidermal thickness, fibrosis in the dermis, and accumulation of inflammatory cells in the dermis. Notably, bvPLA2 treatment induced a dramatic decrease in epidermal and dermal hyperplasia (Figure 3). However, bvPLA2 treatment in CD206^−/−^ mice showed no histological changes compared with AD in CD206^−/−^ mice (Figure 3). In addition, epidermis and dermis thickness were not affected by bvPLA2 treatment in CD206^−/−^ mice (Figure 3B). Similarly, macrophage infiltration of bvPLA2 in WT reduced compared with AD in WT. However, bvPLA2 treatment in CD206^−/−^ mice did not changed macrophage infiltrations in ear tissues (Figure 4).

### 2.6. bvPLA2 Blocked Mast Cell Infiltration in DFE/DNCB-Induced AD via CD206

**Figure 5:**
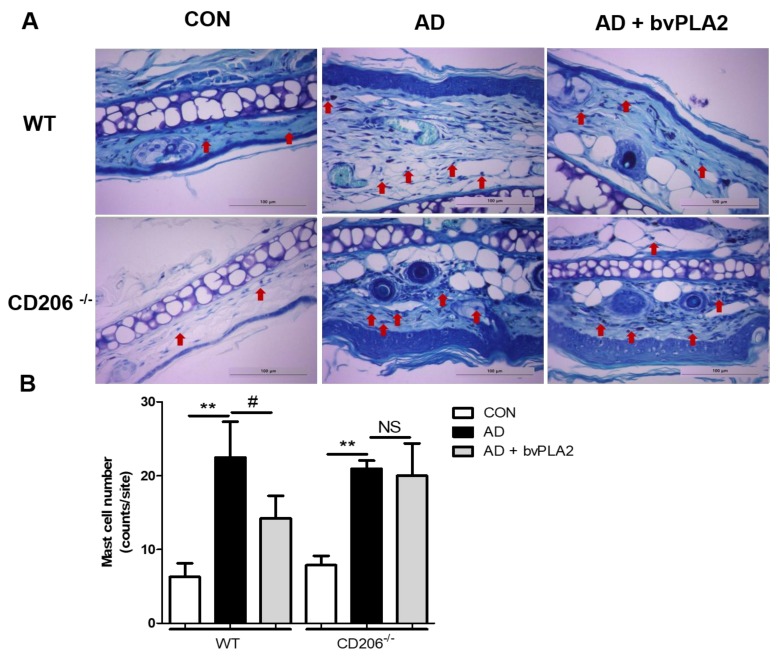
Inhibitory effect of bvPLA2 on DFE/DNCB-induced mast cell infiltration in skin lesions depending on CD206. Mast cell infiltration in skin lesions from wild-type (WT) (**A**) or CD206-deficient (CD206^−/−^) mice (**A**) was evaluated by staining with toluidine blue (TB) (magnification ×200). Mast cell number counts graph (**B**). bvPLA2 was used to treat WT or CD206^−/−^ mice exposed to DFE and DNCB to study whether the suppression of AD-like skin lesions was dependent on CD206. Arrows indicate infiltrated mast cells. CON: normal control, AD: DFE/DNCB treatment, and AD + bvPLA2: DFE/DNCB treatment + bvPLA2 treatment. (** *p* < 0.01 versus Control; NS, ^#^ *p* < 0.05, NS versus AD).

DFE/DNCB treatment induced apparent mast cell infiltration both in wild-type (WT) and CD206-deficient (CD206^−/−^) mice compared with control group as shown in Figure 5. bvPLA2 treatment of WT mice, but not CD206^−/−^ mice, suppressed the increase in mast cell infiltration, as shown by histological analysis of mast cells with toluidine blue (TB) staining (Figure 5) suggesting that bvPLA2 played an anti-inflammatory role through CD206 receptor. In this study, we observed a strong inhibitory effect of bvPLA2 on mast cell infiltration into AD-induced skin lesions (Figure 5).

## 3. Discussion

AD is one of the most common inflammatory skin diseases of unknown etiology [23]. The disease is characterized by the induction of Th2 immune response and IgE hypersensitivity, and symptoms of pruritus and eczematous skin lesions [24]. Although it is still controversial, it is generally known that AD is triggered immunologically by food, aeroallergens, and *Staphylococcus aureus* [25]. Since childhood diseases such as allergic rhinitis, allergic dermatitis, and asthma are closely related to AD, AD patients are strongly susceptible to these diseases [26].

Our previous studies showed that bvPLA2 effectively alleviated AD-like skin lesions in DFE/DNCB-treated mice, suggesting the therapeutic effect of bvPLA2 on AD [10]. In this study, we investigated the cellular metabolism underlying this protective effect of bvPLA2 using a murine model of AD. We found that bvPLA2 (1) comprehensively decreased epidermal and dermal thickness; (2) inhibited upregulation of IgE level; (3) suppressed inflammatory cytokines; (4) increased Treg and (5) blocked infiltration of macrophage and mast cells in AD mice. However, these protective effects of bvPLA2 on AD-like skin lesions were completely abolished in DFE/DNCB-treated CD206-deficient mice suggesting that the benefit of bvPLA2 in AD was mediated via interaction with CD206 mannose receptor. PLA2, a lipolytic enzyme that cleaves the sn-2 acyl bond of the phospholipid, is classified into 15 distinct groups and four main types, depending on their structure [27]. We previously compared PLA2s from various sources in Treg inducing effects and found that PLA2 from bee venom was the most effective among others from cow, pig and snake (unpublished data). Thus, we suggest that an increase in Treg is a specific effect of bee venom PLA2.

It is known that the acute phase of AD is mediated via secretion of Th2 cytokines (IL-4, IL-10, and IL-13) and chronic AD via the secretion of Th1 cytokines (IFN-γ, and IL-6) [28,29]. IL-4 plays a key role in Th2 cell differentiation, IgE synthesis, and eosinophil recruitment. IL-13 also induces isotype switching to IgE synthesis. IL-13, which acts in both acute and chronic stages, also induces isotype switching in IgE synthesis [30]. Total IgE overexpression in serum is one of the major characteristics of AD. In our previous study, we demonstrated that bvPLA2 decreased the expression of serum total IgE in ovalbumin-induced asthma mice model [22]. We also observed a strong inhibitory effect of bvPLA2 on Th1 and Th2 cytokine production in DFE/DNCB-induced Balb/c mice. Despite advances in the development of drug targets in AD, many patients still experience skin challenges mostly due to drug-induced side effects [10].

In this study, we demonstrated a strong inhibitory effect of bvPLA2 on AD-like skin lesions in a murine mice model. However, in the CD206-deficient mouse model, bvPLA_2_ displayed no significant effects on AD-like skin lesions including regulation of serum IgE and inflammatory cytokines. Our histological findings (morphological changes and macrophage infiltration) and mast cell infiltration results also demonstrated the effects in WT mice, but not in CD206-deficient mice, with AD-like skin lesions.

In this study, we investigated the effects of bvPLA2 on AD-like skin lesions using a C57BL/6 mouse model treated with DFE/DNCB. Treatment with bvPLA2 showed significant anti-inflammatory effects on AD-like skin lesions, which were absent in CD206-deficient mice. The study elucidated some of the cellular mechanisms underlying the role of bvPLA2 in the pathogenesis of AD.

## 4. Materials and Methods

### 4.1. Animal

Male 7 to 8-week old C57BL/6 and CD206^−/−^ (B6.129P2-^Mrc1tm1Mnz^/J, Stock No: 007620) mice were purchased from The Jackson Laboratory (Bar Harbor, ME, USA). All mice were housed under pathogen-free conditions with air conditioning on a 12-h light/dark cycle with free access to food and water during the experimental period. All of the experiments were performed in accordance with the Animal Care and Guiding Principles for Experiments Using Animals, and the University of Kyung Hee Animal Care Committee approved this study (KHUASP (SE)-16-073). Date of approval: 30.08.2016

### 4.2. Reagents

AD was induced using American house dust-mite in the form of freeze-dried crude DFE (Greer Laboratories, Lenoir, NC, USA) and DNCB (Sigma-Aldrich, St. Louis, MO, USA) as a sensitizer. The DFE was dissolved in PBS containing 0.5% Tween 20 and the DNCB was dissolved in acetone and olive oil (AOO) in a 3:1 ratio. The bvPLA2 was obtained from Sigma-Aldrich and dissolved in phosphate buffered saline (PBS).

### 4.3. Experimental Protocol

**Figure 6:**
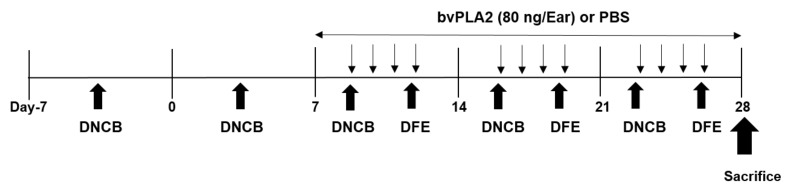
Schedule of experiment. Schematic of experimental design for the induction of atopic dermatitis (AD) in mice. One week after first boosting with 1% 2,4-dinitrochlorobenzene (DNCB), 1% DNCB (20 µL/ear) and *Dermatophagoides farinae* (DFE) (10 mg/mL, 20 µL/ear) was applied alternately to both ears once a week during 4 weeks. The bee venom phospholipase A2 (bvPLA2) (80 ng/ear) was topically applied four times a week for 3 weeks. On day 28, the mice were sacrificed for further analysis.

All experimental protocols were performed in this study as previously described with some modification [10,22,31]. Briefly, the mice were divided into 3 groups (*n* = 3–5 per group). For the induction of atopic disease, the skin of inner ear lobe and outer ear lobe were removed using depilation lotion and surgical tape (Nichiban, Tokyo, Japan). A DNCB solution (20 µL 1%) dissolved in AOO was used in each ear, and 20 μL DFE (10 mg/mL) was then coated for the next 4 days. Over a period of 4 weeks, DNCB and DFE were repeatedly applied for AD induction in the ear. The mice were treated with bvPLA2 four times daily for 3 weeks. The ear thickness was measured twice a week using a dial gauge (Kori Seiki MFG, Co., Tokyo, Japan). On day 28, the mice were sacrificed, and the blood samples and the ear tissues were collected for further analysis. A schematic experimental protocol is depicted in below (Figure 6).

### 4.4. Measurement of Serum Immunoglobulin E (IgE)

For serum total IgE determination, the serum from the mice was analyzed. On day 28, the blood samples were collected from mice and centrifuged at 300 × *g* for 15 min to separate the serum. According to the manufacturer’s instructions, the total serum IgE titers were determined using a mouse IgE ELISA Set (BD Pharmingen, San Diego, CA, USA, Sandwich enzyme-linked immunoassay kit). Briefly, a 96-well microtiter plate (Costar, NY, USA) was coated with 100 µL per well of capture antibody (anti-mouse IgE monoclonal antibody) diluted in coating buffer and incubated overnight at 4 °C. After washing three times with PBS containing 0.05% Tween 20 (Sigma-Aldrich), the plates were blocked with an assay diluent (PBS with 10% FBS) for 1 h at room temperature (RT). Next, the serum samples were diluted (1:250) with assay diluent (PBS with 10% FBS) and 100 µL of serum samples were incubated for 2 h at RT. The detection antibody with Streptavidin-Horseradish Peroxidase (SAv-HRP)reagent (secondary peroxidase-labeled biotinylated anti-mouse IgE monoclonal antibody) was incubated in assay diluent for 1 h at RT. Finally, the plates were treated with TMB substrate solution (BD Biosciences, San Jose, CA, USA) for 30 min, and the reaction was stopped via addition of 50 µL of stop solution. The optical density was measured at 450 nm with a microplate reader (SOFT max PRO, version 3.1 software, San Jose, CA, USA).

### 4.5. Assessment of Th1 and Th2 Cytokine Levels in Mouse Ear

According to the manufacturer’s protocols, the levels of IL-4, IL-10, IFN-γ and IL-6 in the ear tissues were determined using a Mouse ELISA kit (BD Pharmingen, San Diego, CA, USA), which is a sandwich Enzyme-Linked Immunosorbent Assay kit). For protein extraction from the ear tissue, each tissue was homogenized in RIPA Buffer (50 mM Tris-HCl, pH 7.4, 150 mM NaCl, 0.25% deoxycholic acid, 1% Nonidet P-40, 1 mM EDTA) in the presence of protease inhbitor cocktail (Roche Diagnostics, Mannheim, Germany). A 96-well microtiter plate (Costar, NY, USA) was coated with 100 µL per well of the capture antibody (anti-mouse IL-4, IL-10, IFN-γ and IL-6 Monoclonal antibody) diluted in coating buffer and incubated overnight at 4 °C. After washing, the plates were blocked with assay diluent (PBS with 10% FBS) for 1 h at RT. The ear tissue samples were diluted (1:5) with assay diluent (PBS with 10% FBS) and 100 µL of serum samples were incubated for 2 h at RT. The detection antibody containing the SAv-HRP reagent (secondary peroxidase-labeled biotinylated anti-mouse IL-4, IL-10, IFN-γ and IL-6 monoclonal antibody) was incubated in the assay diluent for 1 h at RT. Finally, the plates were treated with tetramethylbenzidine (TMB) substrate solution (BD Biosciences) for 30 min, and the reaction was stopped via the addition of 50 µL of stop solution. The optical density was measured at 450 nm with a microplate reader (SOFT max PRO, version 3.1, CA, USA). The total protein concentrations were determined using a BCA kit (Pierce Biotechnology Inc., Rockford, IL, USA). All results were expressed as relative units by normalization with total protein from each group.

### 4.6. Western Blot Assay

Proteins in the ear lysates were separated by SDS PAGE. After protein transfer onto membranes (DOGEN), membranes were blocked in 5% BSA solution for 1 h and treated with the 1st-antibodies (Foxp3, Santa Cruz, sc-31738; 1:1000 and β-actin Santa Cruz; 1:1000) for 12 h at 4 °C. The membranes were incubated with the 2nd antibodies for 2 h after washing in Tris Buffered Saline with Tween 20 (TBST) three times. After washing the 2nd antibodies, the blots were developed using Western blot analysis system (AbClon Inc., Seoul, Korea).

### 4.7. Histological Analysis 

For the histopathological examinations, the ear samples were fixed with 4% paraformaldehyde solution overnight at 4 °C. The ear tissues were dehydrated, embedded in paraffin and cut into 4 μm sections using a rotary microtome. The sections were stained with hematoxylin and eosin (H&E; Sigma, St. Louis, MO, USA) to evaluate epidermal hyperplasia in the skin of mice as well as infiltration of immune cells into the dermis. IHC staining was performed to measure the degree of macrophage infiltration. Ear tissue sections were stained for CD11b. In brief, 0.3% H_2_O-methanol incubated lung tissues were treated with CD11b antibody (abd-serotec, #mca74g) for 12 h at 4 °C. Subsequently, the tissues were treated following avidin-biotin peroxidase method then, the color was stained by DAB (Zymed Laboratories, South San Francisco, CA, USA).

Toluidine blue (TB) staining was performed to measure the degree of mast cell infiltration. Images of the lung tissue sections were acquired using an Olympus BX51 microscope (Olympus, Tokyo, Japan) and quantified using Image Pro-Plus 5.1 software (Media Cybemetics, Inc., Silver Spring, MD, USA).

### 4.8. Statistical Analyses

The statistical analyses of the data were conducted using Prism 5 software (GraphPad Software Inc., La Jolla, CA, USA). The data are presented as the means ± SD. The differences between the study groups were determined using one-way ANOVA followed by Newman–Keuls multiple comparisons tests. *p* < 0.05 was considered statistically significant.
